# Serial dependence in perception across naturalistic generative adversarial network-generated mammogram

**DOI:** 10.1117/1.JMI.10.4.045501

**Published:** 2023-07-04

**Authors:** Zhihang Ren, Teresa Canas-Bajo, Cristina Ghirardo, Mauro Manassi, Stella X. Yu, David Whitney

**Affiliations:** a University of California, Berkeley, Vision Science Graduate Group, Berkeley, California, United States; b University of California, Berkeley, Department of Psychology, Berkeley, California, United States; c University of Aberdeen, King’s College, School of Psychology, Aberdeen, United Kingdom; d University of Michigan, Department of Electrical Engineering and Computer Science, Ann Arbor, Michigan, United States; e University of California, Berkeley, Helen Wills Neuroscience Institute, Berkeley, California, United States

**Keywords:** serial dependence, generative adversarial networks, visual search, radiological screening

## Abstract

**Purpose:**

Human perception and decisions are biased toward previously seen stimuli. This phenomenon is known as serial dependence and has been extensively studied for the last decade. Recent evidence suggests that clinicians’ judgments of mammograms might also be impacted by serial dependence. However, the stimuli used in previous psychophysical experiments on this question, consisting of artificial geometric shapes and healthy tissue backgrounds, were unrealistic. We utilized realistic and controlled generative adversarial network (GAN)-generated radiographs to mimic images that clinicians typically encounter.

**Approach:**

Mammograms from the digital database for screening mammography (DDSM) were utilized to train a GAN. This pretrained GAN was then adopted to generate a large set of authentic-looking simulated mammograms: 20 circular morph continuums, each with 147 images, for a total of 2940 images. Using these stimuli in a standard serial dependence experiment, participants viewed a random GAN-generated mammogram on each trial and subsequently matched the GAN-generated mammogram encountered using a continuous report. The characteristics of serial dependence from each continuum were analyzed.

**Results:**

We found that serial dependence affected the perception of all naturalistic GAN-generated mammogram morph continuums. In all cases, the perceptual judgments of GAN-generated mammograms were biased toward previously encountered GAN-generated mammograms. On average, perceptual decisions had 7% categorization errors that were pulled in the direction of serial dependence.

**Conclusions:**

Serial dependence was found even in the perception of naturalistic GAN-generated mammograms created by a GAN. This supports the idea that serial dependence could, in principle, contribute to decision errors in medical image perception tasks.

## 1 Introduction

Clinical diagnosis based on radiographs is not always perfect because of misperceptions and misinterpretations.^1^^,^^2^ Some sources of interpretive error have been identified and characterized; these include search and recognition errors,^3^^,^^4^ cognitive biases,^2^^,^^5^ search satisfaction,^6^^,^^7^ subsequent search misses,^8^^–^^10^ and low prevalence.^11^^–^^17^ However, some other errors in cancer image interpretation are still without explanation.^18^^–^^20^ Thus a great deal of research has been carried out in the last several decades to identify and characterize the sources of these errors in order to mitigate them.

Radiologists often read dozens or hundreds of radiographs in batches,^21^ sometimes looking at several related images one after the other. Their job is to localize the lesions (if present) and then to recognize them by judging their size, class, and so on. A main underlying assumption here is that radiologists’ perceptual decisions about the current radiograph are independent of prior perceptual experience.

Recent theoretical and empirical research suggests that this assumption is not true. For example, the human visual system is characterized by visual serial dependency, a type of sequential effect in which what was previously experienced influences (captures) what is seen and reported at this moment.^22^^,^^23^ Serial dependencies can manifest in several domains, such as perception,^23^^–^^26^ decision making,^27^^,^^28^ and memory,^29^^–^^31^ and they occur with a variety of features and objects, including orientation,^23^^,^^32^ position,^26^^,^^33^ faces,^34^^,^^35^ attractiveness,^36^^–^^38^ ambiguous objects,^39^ ensemble coding of orientation,^32^ and numerosity.^24^^,^^40^ Serial dependence is characterized by three main kinds of tuning. First is feature tuning: serial dependence occurs only between similar features and not between dissimilar ones.^23^^,^^26^^,^^32^^,^^41^ Second is temporal tuning: serial dependence gradually decays over time.^23^^,^^26^^,^^39^ Third is spatial tuning: serial dependence occurs only within a limited spatial window; it is strongest when previous and current objects are presented at the same location, and it gradually decays as the relative distance increases.^23^^,^^26^^,^^33^^,^^42^ In addition, attention is a necessary component for serial dependence.^23^^,^^43^^,^^44^

Because our visual world is stable—objects that were present a moment ago tend to still be present at this moment—we benefit from serial dependence most of the time. This is because it is more efficient to simply recycle perceptual history,^23^^,^^24^^,^^26^ using the past to predict the present. However, this recycling is not always beneficial. When stimuli are randomly ordered or in unnatural situations—such as when the visual world is not autocorrelated or stable—serial dependence can negatively impact perceptual decisions.^23^^,^^34^^,^^45^ For example, visual search in clinical settings, such as reading randomly ordered radiographs or pathology slides, is a striking example in which stimuli may not be autocorrelated. In this case, the past may not be a good predictor of the present, and serial dependence in perceptual decisions would be problematic. In fact, empirical experiments have found that clinicians’ perceptual decisions can be biased toward the previous images that they have seen.^46^^,^^47^

A drawback of previous work^46^^,^^47^ is that serial dependence was measured with unrealistic stimuli, such as random geometric shapes superimposed on a mammogram section [Fig. 2(a)]. Although well-controlled, these images are clearly inauthentic and are therefore far from naturalistic mammograms.^46^^,^^47^ Unfortunately, because serial dependence has only been measured with unrealistic stimuli, it remains unclear whether serial dependence in perceptual judgments would even occur for truly realistic radiographs.

In this study, we aim to measure the presence of sequential effects in the perceptual decisions of observers who view controlled, realistic generative adversarial network (GAN)-generated radiographs. To accomplish this, we created authentic-looking medical images generated by a computer vision model. The model allows precise control over the stimulus space, while simultaneously ensuring that the simulated radiographs are realistic. In fact, a previous study found that these images are indistinguishable from (i.e., metameric with) down-sampled real radiographs, even to many professional clinicians.^48^^,^^49^ We hypothesize that even with authentic-looking simulated mammograms, perceptual decisions about any given current image will be biased toward the previously seen images due to serial dependence.

## 2 Methods

### 2.1 Mammogram Generation

**Fig. 1:**
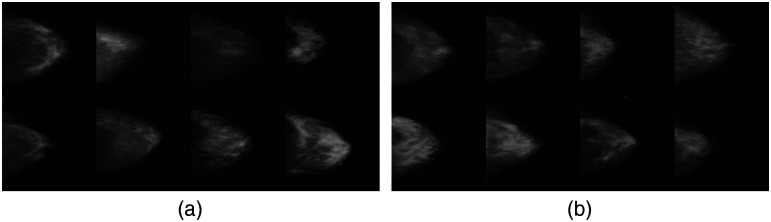
Generated samples via GAN. Here we show a comparison between (a) the real sample (down-sampled mammograms from DDSM dataset that are collected from the hospital) and (b) GAN-generated samples. After training, GAN learns the image manifold of down-sampled real samples and then samples on the learned manifold to generate novel simulated samples. Additionally, because the manifold has been learned, interpolation can be applied to generate quantifiably similar images. The resolution of the real and generated samples is equated.

In computer vision, generative models^50^^,^^51^ have been utilized for authentic image generation for years. In particular, GANs are a promising method to create authentic images in different modalities of human faces, places, animals, cars, etc.^52^^–^^54^ Similar approaches have also been applied for medical image generation.^48^^,^^55^^–^^57^ In this study, we adopted a controllable medical image generation method^48^^,^^49^ to create all stimuli used in our experiments. Because of the GAN generation paradigm, the generated samples share the similar data distribution as the real samples maintaining the variety of the real ones. A comparison between downsampled real samples and generated samples is shown in Fig. 1.

**Fig. 2:**
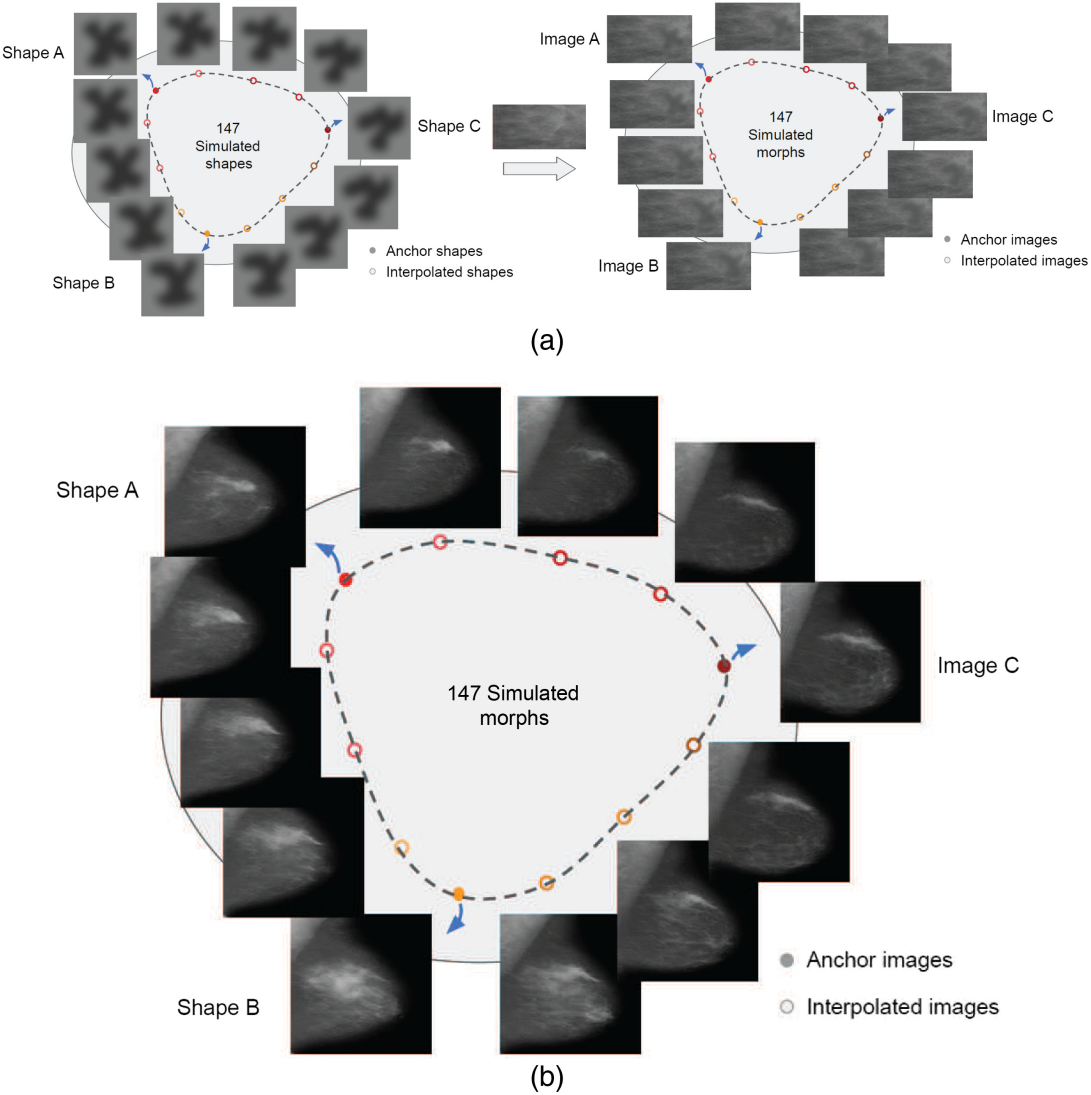
Comparison between stimuli used in previous experiments and current GAN-generated stimuli. (a) Stimuli from previous works.^46^^,^^47^ A circular continuum of simple shapes is generated first, and then each shape is fused onto a mammogram tissue background section to form the experiment stimuli. (b) We randomly picked three anchor points in the latent space (images A, B, and C shown with solid dots] and generated 48 interpolated morphs in between each pair (shown with hollow dots) via GAN (147 morphs in total) to form a circular morph continuum. In total, 20 circular continuums were generated. Here we show 1 continuum as an example. More continuum examples can be found in Fig. 3.

**Fig. 3:**
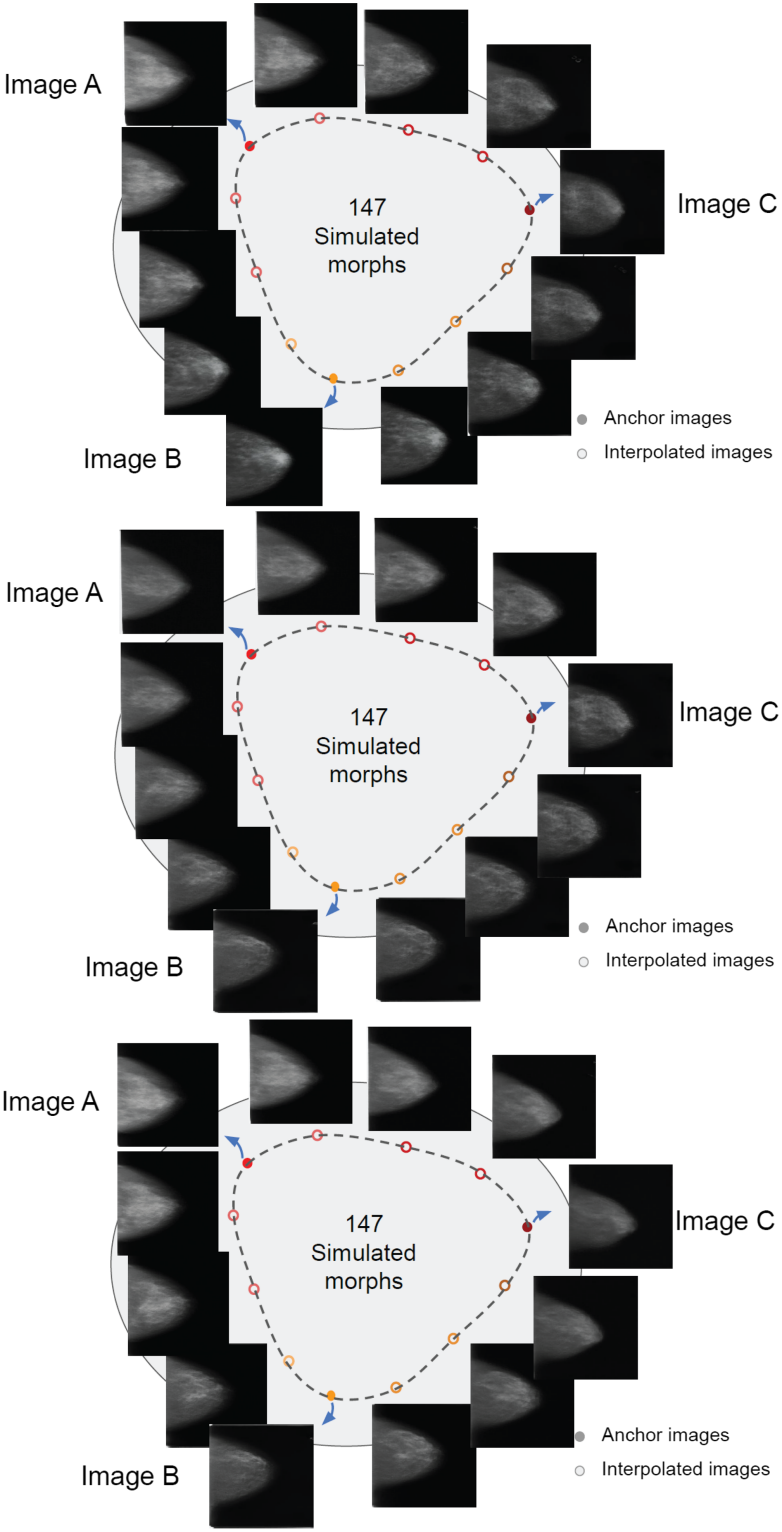
Three extra example continua. Each shows a circular morph continuum generated from different anchor sets. Here we only show 3 out of 48 interpolations between anchor points. The actual similarity steps between sequential interpolations are much closer.

Once the GAN was pretrained, we randomly sampled points on the straight lines connecting every pair of 3 anchor points in the latent space, and generated the images corresponding to these points. Each anchor point and the corresponding interpolations are latent vectors with the size of 512 (see the publications^48^^,^^49^ for thorough details about the model and latent space.). Then we passed the anchors as well as the interpolations through the pretrained generator to generate the corresponding images, forming a circular continuum [Fig. 2(b)]. One hundred and forty-seven images (48 between each anchor) were generated on the circular continuum with size 256×256. (The reason we used 256×256 was for proof of concept and because the training takes exponentially longer with higher-resolution images.). In the experiment, 20 circular continuums such as this were generated by creating 20 sets of anchors and passing them along with the corresponding interpolations to the pretrained generator. In total, we generated 2940 images. Four example continua are shown in Fig. 3.

Because the images within a given continuum were generated based on interpolations in the latent space, nearby images on the circular continuum tend to be more similar, whereas distant images on the circular continuum tend to differ from each other. With random picking, the generated image sequence can represent a certain variety of the real samples. Moreover, moving around the circular continuum, the tissue texture, tumor size, tumor location, and other semantic properties gradually change and return to the same place when looping through all of the GAN-generated mammograms on this circular continuum.

### 2.2 Dataset

Training data for the GAN are from the digital database for screening mammography (DDSM).^58^ It contains 2620 normal, benign, and malignant cases with verified pathology information. The images were first center cropped and then resized to 256×256 for training. To generate stimuli containing tumors for the visual search task, only benign and malignant cases were utilized for training. Several downsampled real samples are shown in Fig. 1.

### 2.3 Participants and Apparatus

All experimental procedures were approved by and conducted in accordance with the guidelines and regulations of the UC Berkeley Institutional Review Board. Participants provided informed consent in accordance with the IRB guidelines of the University of California at Berkeley. All participants had normal or corrected-to-normal vision and were all naïve to the purpose of the experiment. 80 nonexpert participants (28 males, aged 18 to 72, and 52 females, aged 18 to 62) participated in the experiment. They were students and affiliates at UC Berkeley.

Experiments were coded with PsychoPy and published on Pavlovia. Participants were able to access the experiment by themselves through the Internet. Sets of 4 participants were assigned to the same circular continuum, and there were 20 circular continuums in total (for a total of 80 observers). Participants used a keyboard for all responses.

### 2.4 Experiment Design

**Fig. 4:**
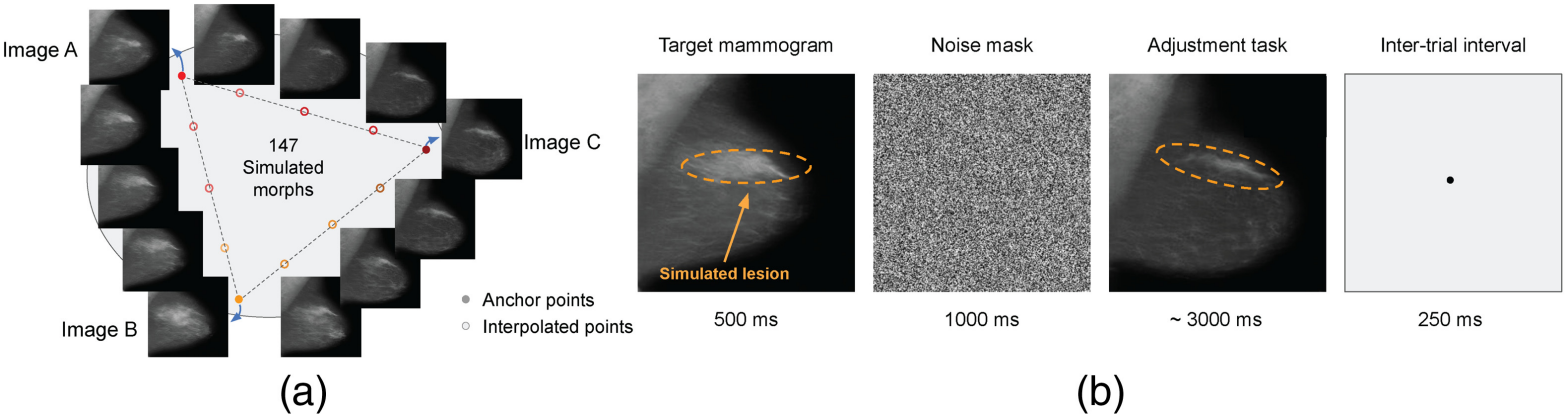
Stimuli and experiment design. (a) An example circular continuum generated via GAN. (b) Observers were presented with a random morph on a specific morph continuum, followed by a noise mask. They were then asked to adjust the morph (the start point is randomly picked along the same morph continuum.) to match the target morph that they previously saw and to press space bar to confirm. During the inter-trial interval, a black fixation dot appeared in the center. After a 250 ms inter-trial interval, the next trial started.

The 20 circular morph continua mentioned in Sec. 2.1 were used to test the perceptual decisions of the participants. Each simulated mammogram of any continuum contains a particular pattern of lesions and texture, and these characteristics gradually change along the circular continuum. On each trial, participants viewed a random simulated mammogram, which was randomly extracted from one of the 20 circular continua, mimicing the randomness in real diagnostic scenarios. The simulated mammogram was presented for 500 ms. Next, we presented a mask composed of random Gaussian noise for 1000 ms (to avoid the possibility of afterimages). After the mask, a random simulated mammogram drawn from the same morph continuum appeared at the fixation point location, and participants were asked to adjust the simulated mammogram to match the perceived simulated mammogram using the left/right arrow keys (continuous report, adjustment task; left–right arrow keys to adjust the simulated mammogram along the circular morph continuum). The starting simulated mammogram was randomized on each trial. Participants were allowed to take as much time as necessary to respond and pressed the space bar to confirm that the chosen simulated mammogram was the correct match. Following the response and a 250 ms delay, the next trial started. A concise experiment pipeline can be found in Fig. 4(b).

During the experiment, participants were asked to continuously fixate a black dot in the center. In total, each participant performed 3 blocks of 85 trials. Between each block, participants were allowed to take a break. In a preliminary session, observers completed a practice block of 10 trials to familiarize themselves with this experiment. Among the 80 participants, 3 participants were removed from data analysis because they hit the space bar all of the time during the experiment without any adjustments.

### 2.5 Data Analysis

The response error was computed as the smallest difference along the morph continuum between the match morph and the target morph (current match morph–current target morph). For each participant’s data, trials were removed if the response error was 3 standard deviations away from the mean response error or if the response time was longer than 20 s. The average reaction time was 3.42±2.47  s.

Previous research shows that individual observers can have idiosyncratic biases in object recognition and localization, which are unrelated to serial dependence.^59^^,^^60^ For example, observers may make a consistent error in reporting a simulated lesion of 20 morph units as being 10, thus creating a systematic error of −10  morph units. Conversely, if there was no systematic error, all error would approximate zero. For this reason, we conducted an additional data processing strategy to remove such potential unrelated biases before further analyses. We modeled observers’ response error as a function of the target morph presented by fitting a radial basis function in which 30 Gaussian kernels are utilized. This allowed us to quantify the idiosyncratic bias for each observer. We then regressed out the bias quantified by the radial basis fit by subtracting it from the observer’s error. This subtraction left us with residual errors that did not include the idiosyncratic biases unrelated to serial dependence.

#### 2.5.1 Feature tuning analysis

The difference in morphs between the current and previous trial is computed as the smallest difference along the morph continuum between the previous target morph (n-back) and the current target morph (previous target morph–current target morph). To quantify the feature tuning characteristic of serial dependence, we fit a derivative of von Mises distribution to each observer’s data points. The derivative of von Mises distribution is expressed by the following equation:

$$y=-\frac{a\kappa \;\sin(x-\mu )e^{\kappa \;\cos(x-\mu )}}{2\pi I_{0}(\kappa )},$$ (1)

where parameter y is the response error on each trial, x is the relative orientation of the previous trial, a is the amplitude modulation parameter of the derivative-of-von-Mises (DoVM), μ is the symmetry axis of the derivative of von Mises distribution, κ is the concentration of the derivative of von Mises distribution, and $I_{0}(\kappa )$ is the modified Bessel function of order 0. In our experiments, μ is set to 0. We fitted the derivative of von Mises using constrained nonlinear minimization of the residual sum of squares. As a measure of serial dependence, we reported half the peak-to-trough amplitude of the DoVM.

**Fig. 5:**
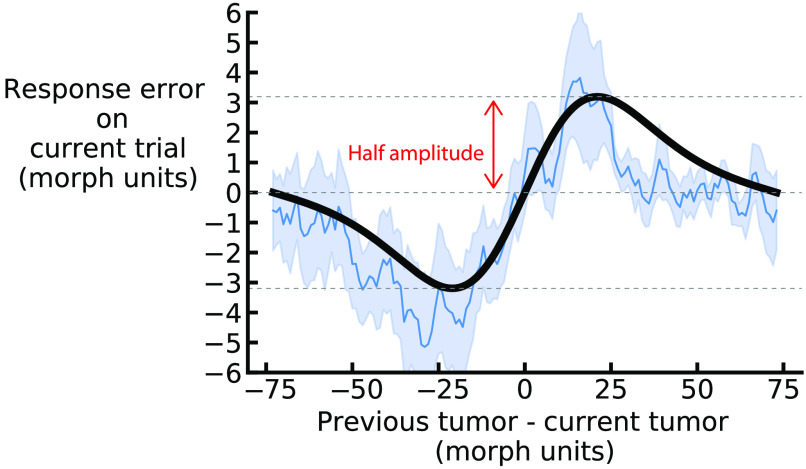
DoVM curve fit for a representative continuum (one of the twenty different morph continuums. In units of shape morph steps, the x axis is the shortest distance along the morph continuum between the current and one-back simulated lesion, and the y axis is the shortest distance along the morph continuum between the selected match shape and current simulated lesion. Positive x axis values indicate that the one-back simulated lesion was clockwise on the shape morph continuum relative to the current simulated lesion, and positive y axis values indicate that the current adjusted shape was also clockwise relative to the current simulated lesion. The average of the running averages across observers (blue line) reveals a clear trend in the data, which followed a DoVM shape (model fit depicted as black solid line; fit on average of running averages). Light-blue shaded error bars indicate the standard error across observers. We operationalized the strength of pull toward the previous observed stimuli as the half amplitude of the derivate-of-von-Mises curve, as noted in red.

Additionally, for each observer, we computed the running circular average within a 20 morph units window. Figure 5 (blue line) shows the average of the moving averages across all observers and the corresponding DoVM fit.

#### 2.5.2 Temporal tuning analysis

In this study, we report half the peak-to-trough amplitude of the DoVM as a measure of serial dependence (Fig. 5). Sequentially, we can get the strength of 1-back, 2-back, and 3-back serial dependence effects by fitting the derivative of von Mises distribution on the data points, where the difference in morphs between the current and previous trial is computed as the smallest difference along the morph continuum between the 1-, 2-, and 3-trial back target morph and the current target morph.

Additionally, as a control analysis, we explored the effect of future trials on the current response to check for potential unrelated biases and artifacts that might be lurking in the data.^30^^,^^61^ In particular, we calculated whether the current trial response error depended in some fashion on the difference in stimuli between the current and 1-forward (following) trials. Because observers have not seen the future trial stimulus, their current response in a given trial should not be influenced by the future morph stimuli. If there are artifacts in the data, however, (for example, observers perseverate on a particular response from trial to trial), there might appear to be an effect of future stimuli on the current response. This analysis reveals and serves as a control for such artifacts.^23^^,^^47^

##### Bootstrapping

For each result that we obtained, we resampled the data with replacement, processed the sampled data recursively for 5000 times, and reported the mean result with 95% confidence intervals.

##### Permutation test

Significance testing was done through permutation tests. Data were randomly shuffled and processed 5000 times. The 97.5% upper bound of the permuted null distribution was compared with the error bar from bootstrapping to confirm the significance of the result.

In an additional analysis, to more intuitively convey the magnitude of the serial dependence effect, we analyzed the percentage difference between pro-SD (pulling effect due to serial dependence) and anti-SD (repelling effect against serial dependence) for 1, 2, and 3 trials back. Stimuli on the circular continuum were categorized into three types according to the nearest anchor images. Trials in which the response image was not within the same category as the target image were considered classification errors, which are misjudgments of the image category. Classification errors that are in a direction consistent with the previously seen stimulus are pro-SD errors, and those that are in a direction opposite the previously seen stimulus are anti-SD errors. In principle, classification errors should be randomly distributed, not biased in either direction. As a sanity check, we also analyzed the percentage difference between pro-SD and anti-SD for 1 trial forward because future trials naturally are not correlated with current trials.

## 3 Results

The goal of this experiment was to test whether perceptual decisions on consecutive realistic GAN-generated images of mammograms were biased toward the previously seen images. Here the observers’ response error in a particular trial was computed as the shortest distance along the morph continuum between the actual observed shape and the chosen answer shape. The average response error was 17.26±5  morph units, and the average reaction time was 3.43±1.50  s.

To test whether there are sequential effects in observers’ judgments of realistic GAN-generated mammograms, we first analyzed the response error in relation to the difference in stimulus shape between the current and previous trials for each continuum separately. Then we fitted a DoVM function to this data (Fig. 5).

**Fig. 6:**
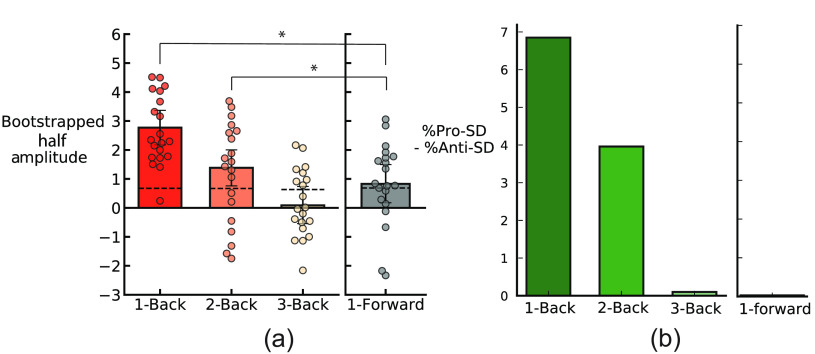
(a) Bootstrapped half amplitudes of DoVM fit for 1, 2, and 3 trials back. Half amplitude for 1-forward is shown as a comparison (gray bars). Each filled dot represents the bootstrapped half amplitude for a single circular morph continuum. Bars indicate the group bootstrap, and error bars are bootstrapped 95% confidence intervals. (b) Classification error analysis. Stimuli on the circular continuum are categorized into three types according to the nearest anchor images. Classification errors are categorized based on distance to the three anchors. Pro-SD means the classification error on the current trial is attracted toward the previous stimuli, whereas anti-SD means the current classification error is repelled from (opposite) the previous stimulus. The differences in these two types of error are computed for 1, 2, and 3 trials back and for 1 trial forward as a control.

We operationalized serial dependence, the pull toward previous stimuli, as the half amplitude of the DoVM curve of each continuum. We bootstrapped the half amplitude and reported the average bootstrapped half amplitude for each continuum: all continua showed a positive half amplitude [Fig. 6(a)]. Importantly, the average half amplitude across all continua was significant (average bootstrapped 1-back half amplitude = 2.77 morph units, p<0.001, permutation analysis), which suggests an influence of the simulated radiograph in the previous trial on the current response. The influence of previous stimuli extended to two trials back (average bootstrapped 2-back half amplitude = 1.38 morph units, p<0.01, permutation analysis). By contrast, the stimuli presented three trials prior had no influence on the current response (average bootstrapped 3-back half amplitude = 0.09 morph units, p>0.05, permutation analysis). To control for artifacts, we calculated the influence of the stimuli presented in the next trial on the current response. We found a modest bias, as found in previous studies of sequential effects,^61^^,^^62^ but, importantly, the 1-back and 2-back effects were significantly larger than this 1-forward baseline (1-back versus 1-forward: p<0.05; 2-back versus 1-forward: p<0.05. This confirms that there are sequential effects in perceptual decisions about realistic GAN-generated mammograms.

To quantify the serial dependence effect in an alternative manner, we also analyzed the percentage difference between pro-SD and anti-SD classification errors [Fig. 6(b)]. Overall, the classification error rate is 28.35%. The 1-back and 2-back percentage differences were 6.85% and 3.96%, respectively, indicating the dominance of serial dependence in the sequential effects in perceptual decisions of participants. Essentially, when there are classification errors, these are much more likely to be in the direction of previous stimuli. The 3-back percentage difference was 0.1%. Overall, serial dependence dominated the sequential effects for 1 and 2 trials back. In addition, the sanity check of 1 trial forward, 0.03%, shows no influence of future trials on classification errors in the current trial. This is expected and confirms that there were no artifacts masquerading as serial dependence.

## 4 Discussion

Serial dependence in medical image perception has been studied for years.^46^^,^^47^ However, none of the previous research used realistic medical images. In previous studies, the stimuli incorporated simple geometric shapes and artificial backgrounds consisting of either healthy tissue texture or simple noise patterns. Although the prior empirical results indicate the existence of serial dependence in the perception of those unrealistic stimuli, whether serial dependence extends to and occurs for realistic medical images remained unknown.

In this study, we tested whether there is serial dependence in perceptual judgments of more realistic GAN-generated radiographs. We utilized authentic-looking simulated medical image stimuli created with a GAN.^48^^,^^49^ The magnitude of serial dependence found in the current study was similar to that found in previous studies. Prior studies found that perceptual judgments were pulled toward the stimulus presented in the previous trial, and the pull effect was around 15% for 1-back trials. Moreover, this effect lasted up to 10 s or more in the past.^47^ The results in this study were comparable. For example, the half amplitudes of the DoVM curve in Fig. 6 show a similar effect size as that previously reported. This indicates that serial dependence affects untrained observers’ judgments of the simulated radiographs. The fact that clinicians show serial dependence in other domains,^46^^,^^47^ and the fact that serial dependence can increase with expertise^63^ hints at the possibility that clinicians may not be immune from serial dependence. Nevertheless, whether serial dependence influences clinician judgments of the more realistic GAN-generated radiographs here remains an important question for future research.

In addition to replicating and extending the presence of serial dependencies in perceptual judgments of realistic medical images, our study also highlights the broader point that computer vision tools can be used in concert with psychophysical experiments to isolate and shed light on human performance limits. Computer vision models, in this approach, are not employed with the goal of replacing human readers. Rather, computer vision is used to create controlled stimuli that allow human performance to be more accurately assessed, controlled, and potentially enhanced. Computer vision models are in the service of human behavior.

There are several caveats and concerns that readers may have noted. It may be argued, for example, that the presentation duration of the simulated mammogram was too short (500 ms) or too low resolution (256×256) in our study, whereas clinicians typically have longer periods of time to process higher-resolution radiographs. In fact, the average fixation duration when targeting the first mass has been reported as 1.8 to 2 s, which is surprisingly brief.^4^^,^^64^ Moreover, when scrolling through volumetric images, the viewing time in any given slice can be a fraction of a second. In addition, peripheral viewing and effectively lower resolution images can be sufficient for detecting abnormalities.^15^^,^^65^^,^^66^ Conversely, images viewed for a sufficiently long exposure duration can lead to negative aftereffects. For example, it was found that adapting normal observers to image samples of dense or fatty tissues caused a subsequent image to appear less dense (and vice versa; a type of negative aftereffect).^67^^–^^69^ Sequential effects (either repulsive or attractive) can therefore emerge across many different exposure durations.

In addition to the fixed duration of the stimuli in this experiment, this study has some additional limitations. First, we chose a continuous report matching task in our experiments as it provides precise trial-wise errors and has proven to be very reliable in measurements of serial dependence in the past.^23^^,^^24^^,^^34^^,^^41^^,^^43^^,^^70^ However, the actual task of the typical radiologist is far more complicated and involves detecting, locating, and classifying the lesions. Future studies should therefore implement more realistic tasks. Second, we only tested untrained observers in this study. Future studies should also recruit clinician observers. Third, the simulated mammograms were only presented briefly in our experiment to mimic the brevity of images viewed in quick succession. To generalize the results here, it will be necessary to test which biases arise with longer presentation durations. Fourth, even though we utilized both benign and malignant images for training, we did not consider the malignancy of the stimuli in the GAN model and experiments. Future studies can investigate how malignancy can be disentangled in the GAN model and how malignancy may influence the diagnostic tasks. Our goal in this study was to test the presence of sequential effects in judgments of more realistic and controlled GAN generated medical images, and we found evidence for this. However, the caveats and concerns described here prevent us from concluding that serial dependence impacts clinical image interpretation in real clinical practice. The results raise the possibility, though, and if there are serial dependencies in clinical interpretations, then the consecutive similarity between images from one or more patients could matter. Future work is needed to test this.

## 5 Conclusion

In this study, we utilized a GAN to produce authentic-looking GAN-generated mammograms. These realistic stimuli were used in a psychophysical experiment that tested for serial dependence in perceptual judgments. We found that the perception of the current simulated mammogram was biased toward the previously seen mammograms. On average, perceptual judgments of naturalistic GAN-generated mammograms had 7% categorization errors that were pulled in a direction consistent with serial dependence, and this pulling effect lasted up to 10 s in the past. Our study provides evidence that serial dependence may contribute to the decision errors in the perception of realistic-looking medical images.
